# Occult Acetabular Fracture Associated With Periprosthetic Femoral Fracture (Vancouver Type B3) Following Hemiarthroplasty in an Elderly Patient: A Case Report

**DOI:** 10.7759/cureus.72071

**Published:** 2024-10-21

**Authors:** Toshiaki Haraguchi, Shinichiro Kume, Kotaro Jimbo, Koji Hiraoka, Takahiro Okawa

**Affiliations:** 1 Department of Orthopedic Surgery, Kurume University Medical Center, Kurume, JPN; 2 Department of Orthopedic Surgery, St. Mary’s Hospital, Kurume, JPN; 3 Department of Orthopedic Surgery, St. Mary's Hospital, Kurume, JPN; 4 Department of Orthopedic Surgery, Kurume University School of Medicine, Kurume, JPN

**Keywords:** acetabular fracture, hemiarthroplasty, periprosthetic femoral fracture, postoperative leg pain, revision hip surgery

## Abstract

With the increase in life expectancy, the number of elderly individuals undergoing hemiarthroplasty and total hip arthroplasty has risen, leading to a higher incidence of implant-related fractures. Diagnosing fractures, especially occult and non-displaced ones, can be challenging even with advanced imaging techniques. This report describes the diagnostic challenges and surgical management of a rare combination of periprosthetic femoral fracture and an ipsilateral acetabular occult non-displaced fracture. An 87-year-old woman with a history of left hemiarthroplasty experienced severe left hip pain after a fall and required an ambulance. Despite computed tomography evaluations, preoperative diagnosis of a left periprosthetic femoral fracture with stem loosening and ipsilateral acetabular occult non-displaced fractures was challenging. Intraoperatively, a non-displaced acetabular fracture with severe bone fragility was unexpectedly found. The surgical procedure involved inserting a revision femoral stem to bypass the fracture site and performing osteosynthesis for the acetabular fracture. Two years postoperatively, the patient’s activities of daily living improved to the preoperative level. Periprosthetic femoral fractures combined with acetabular fractures can occur following hemiarthroplasty and total hip arthroplasty, presenting significant diagnostic challenges in preoperative imaging evaluations. Surgeons should always consider the possibility of acetabular fractures when planning revision total hip arthroplasty for periprosthetic femoral fractures and be prepared to address them appropriately.

## Introduction

In recent years, with the increase in life expectancy, the number of elderly individuals undergoing procedures, such as hemiarthroplasty (HA) and total hip arthroplasty (THA), has increased. In the United States, in 2013, more than 1 million hip and knee arthroplasties were being performed each year and that number has been continually rising.

Consequently, the incidence of implant-related fractures has also increased and is expected to continue rising [1]. Between 2006 and 2014, periprosthetic fracture emerged as the fastest-growing cause of primary THA failure and an indication for revision of THA. This growth surpassed that of all other indications, including periprosthetic joint infection, aseptic loosening, osteolysis, instability, implant failure, and bearing surface wear. Specifically, the relative proportion of revision THAs attributed to periprosthetic fracture increased by 5.3% during this period. From 2016 to 2021, The incidence of periprosthetic hip fractures rose by 38%. The number of periprosthetic hip fractures is predicted to rise 189% from 2016 to 2032 [1,2].

The incidence of periprosthetic fracture associated with THA varies, with many studies reporting an incidence of 0.1%-18% [2,3]. Evidence suggests a relatively high incidence (approximately 2.4%) of periprosthetic femoral fracture (PPFF) following HA, even with a relatively short average follow-up period of 44.4 months [4].

PPFF can occur even after a long period following HA. In these cases, after a long period following HA, progressive bone fragility and cortical thinning due to stress shielding and osteolysis can increase the risk of PPFFs. When these conditions are present, the prosthetic stem may become loose, thereby complicating treatment. In addition, periprosthetic fractures in the elderly present significant surgical challenges due to bone quality. However, such cases are becoming increasingly common in daily clinical practice.

The incidence of occult fractures around the hip joint, including the pelvis, is reported to be 2%-10% and that specifically of acetabular fractures is much lower at 0.35%-0.44%, indicating a very rare occurrence [5]. While proximal femur fractures combined with acetabular fractures are occasionally reported in high-energy trauma cases, to the best of our knowledge, a PPFF with stem loosening after HA combined with a non-displaced acetabular insufficiency fracture has not yet been reported. This report describes the diagnostic challenges and surgical management of this rare combination of PPFF with stem loosening after BHA and non-displaced acetabular insufficiency fracture.

## Case presentation

**Figure 1:**
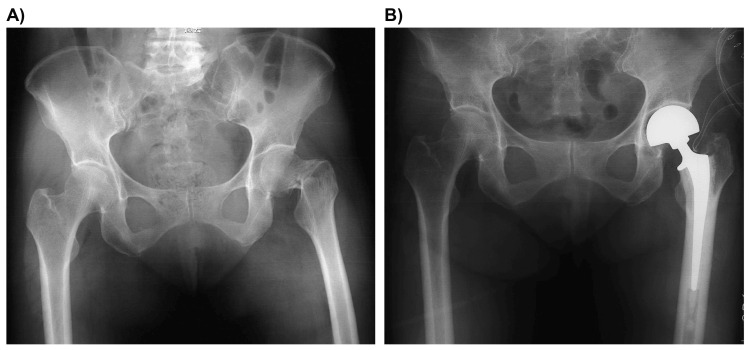
Initial preoperative and postoperative radiographs for hemiarthroplasty of a femoral neck fracture (anteroposterior view). The cemented stem is well-fixed with satisfactory alignment and positioning. (A) Preoperative view. (B) Postoperative view.

An 87-year-old female patient presented to our emergency department by ambulance, presenting with severe left hip pain and swelling after a fall in her yard. She weighed 48.6 kg, stood 150 cm tall, and had a body mass index of 21.6 kg/m². Prior to the fall, her activities of daily living (ADL) included walking with a single cane, and she experienced only mild left thigh pain while walking for a few years. The patient had a history of cemented bipolar HA (BHA) for a displaced femoral neck fracture performed at another hospital when she was 65 years old. She had recovered well postoperatively; however, the details of the surgery were unclear, and she was lost to follow-up thereafter. Postoperative radiographs indicated that the cemented stem was well-fixed with satisfactory alignment and positioning (Figures 1A, 1B). 

**Figure 2:**
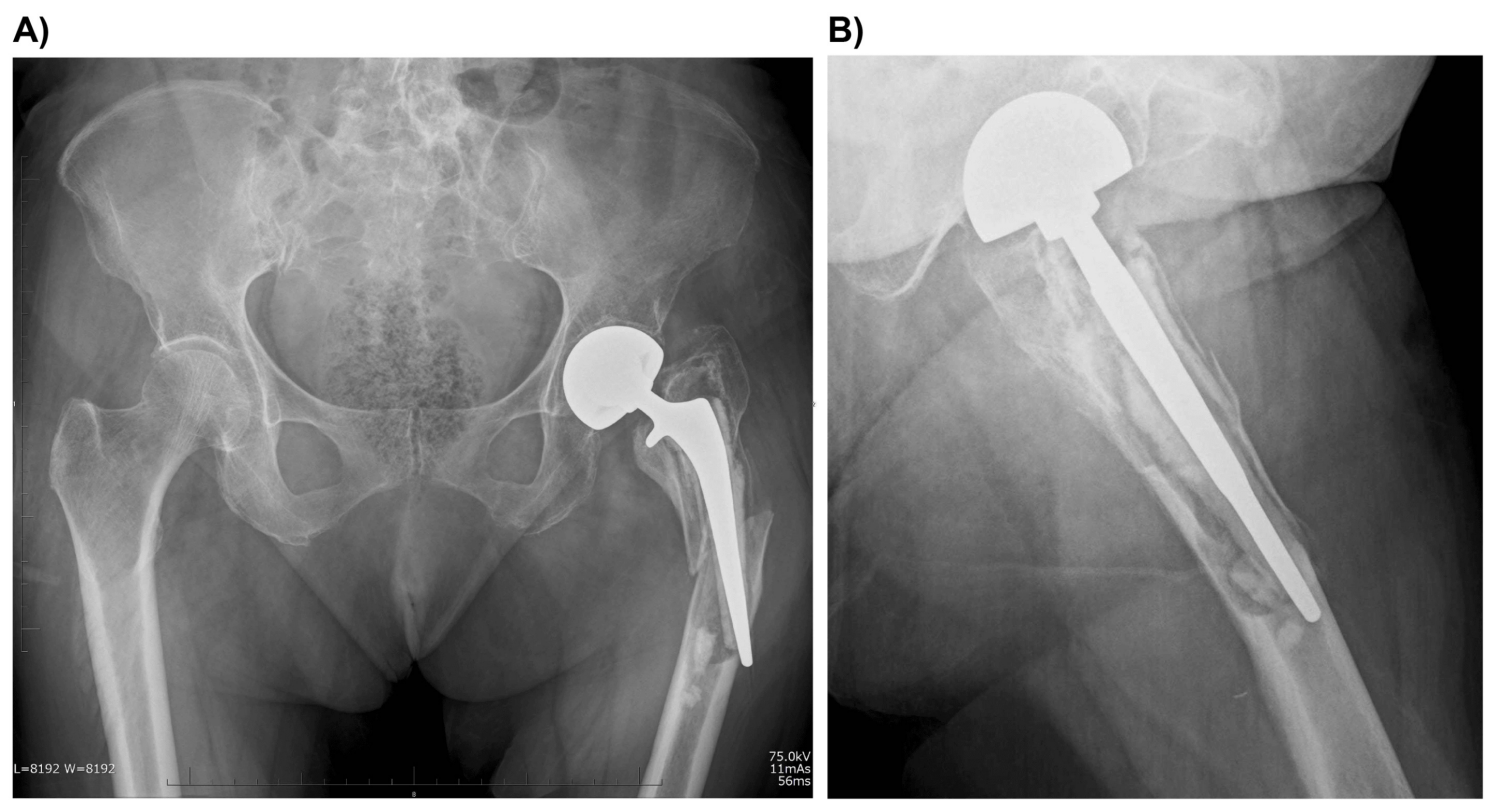
Preoperative radiographs of a periprosthetic femoral fracture showing cement debonding and severe osteolysis. (A) Anteroposterior view. (B) Lateral view.

**Figure 3:**
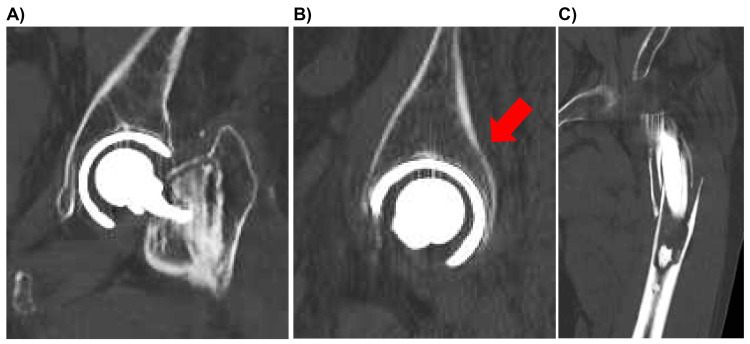
Initial preoperative computed tomography images of the acetabular bone (A, B) and femoral bone (C). (B) Minimal discontinuity of the cortical bone, with no clear acetabular fracture identified (arrows).

Her motor and sensory examinations were unremarkable, and although his pain was severe, it did not correspond to any specific anatomical nerve distribution. Radiographs of the hip joints in the anteroposterior view (Figures 2A, 2B) and computed tomography (CT) (Figures 3A-3C) revealed a PPFF with cement debonding and severe osteolysis, which led to polyethylene wear. The stem was loose, and the surrounding bone stock was inadequate. We diagnosed the fracture as Vancouver classification type B3. There was no clear evidence of an acetabular fracture.

**Figure 4:**
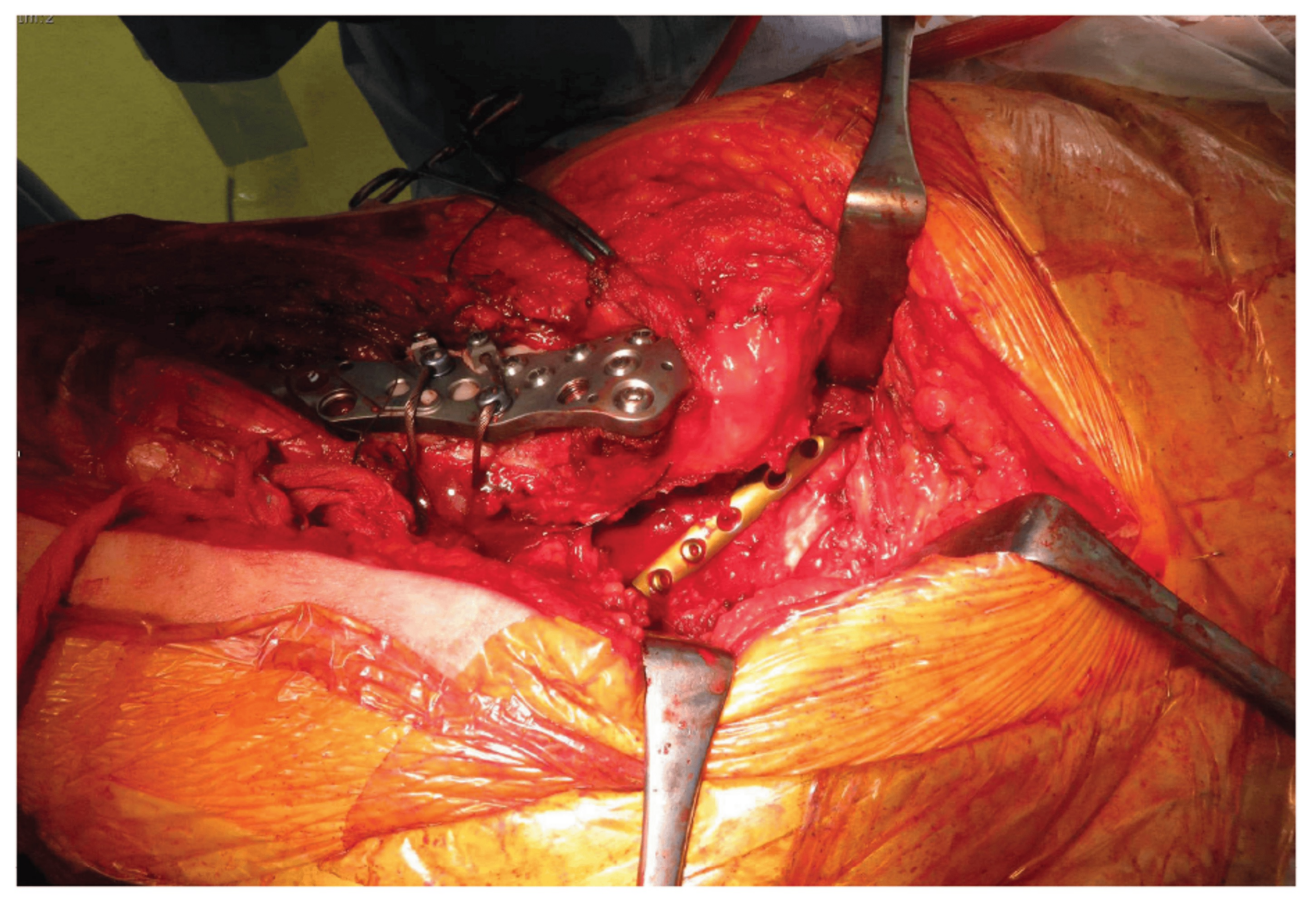
Intraoperative photograph displaying the acetabular fracture after osteosynthesis.

Preoperatively, revision BHA was planned to address the PPFF, with a posterolateral approach in the lateral position. The operation was performed eight days post-injury. During surgery, the revision of BHA was completed, and osteosynthesis for the acetabular fracture was also performed. An unexpected finding was a non-displaced acetabular fracture associated with severe bone fragility (Figure 4).

The cortical bone around the PPFF was markedly thinned and crushed. The cement had debonded and was easily removable. The fracture was repositioned as much as possible and stabilized with a plate. Revision surgery was performed using a cemented long stem.

**Figure 5:**
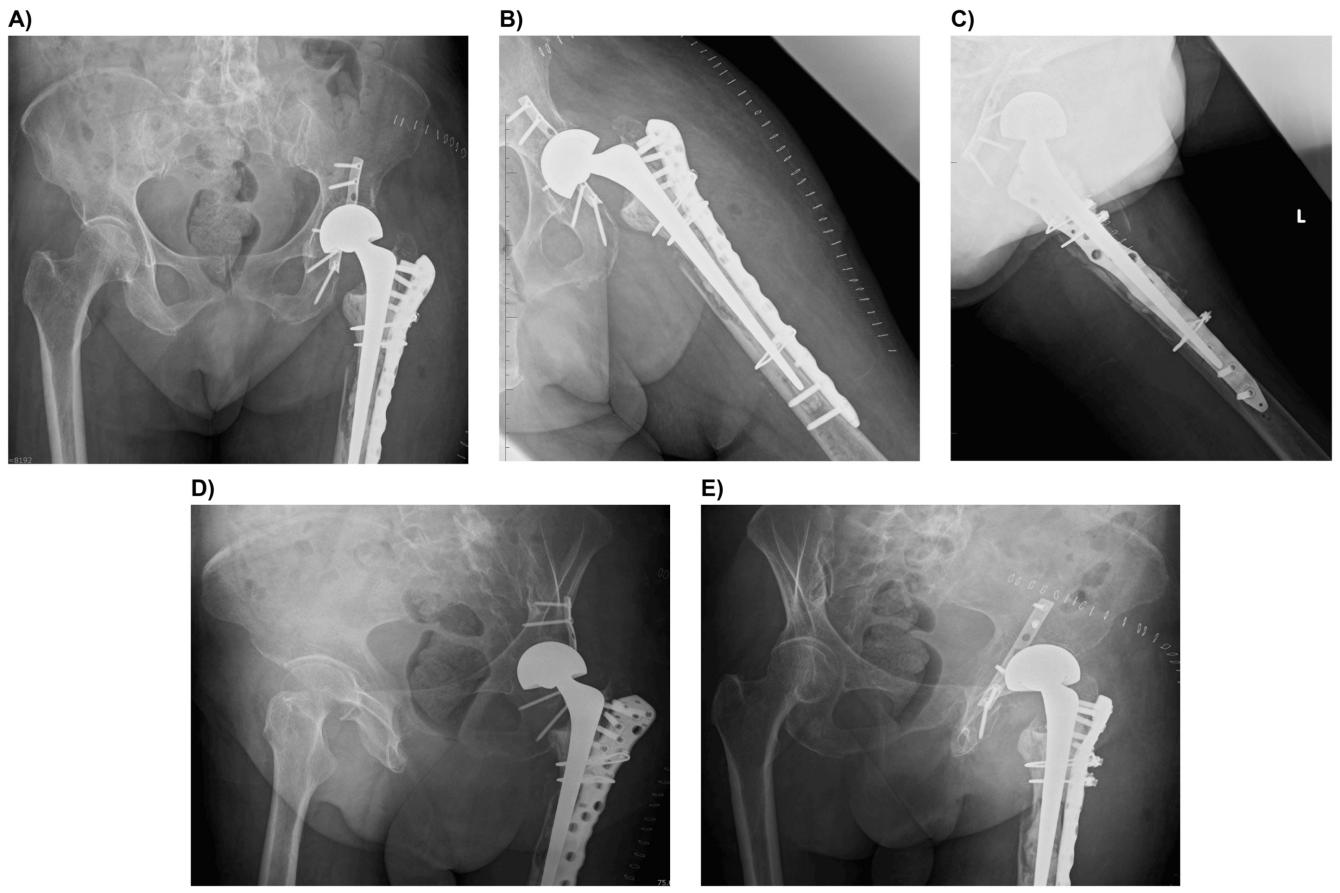
Postoperative radiographs showing the revision procedure performed with a cemented long stem and osteosynthesis. (A, B) Anteroposterior view. (C) Lateral view. (D) Right oblique pelvic view. (E) Left oblique pelvic view.

During the procedure, an examination of the fracture site on the posterior acetabular wall revealed progressive dislocation. A transverse fracture on the posterior wall was identified and classified as a transverse-type acetabular fracture. A one-third plate was used to fix the acetabular fracture to the posterior wall, followed by reduction and internal fixation of the acetabular fracture (Figures 5A-5E).

This resulted in anatomical reduction and alignment without risk factors for failure that might necessitate delayed THA, such as dome or roof impaction and bone defects, and the decision was made to proceed with BHA without acetabular cup implantation. Partial weight-bearing was allowed starting at four weeks postoperatively, with full weight-bearing permitted at eight weeks.

**Figure 6:**
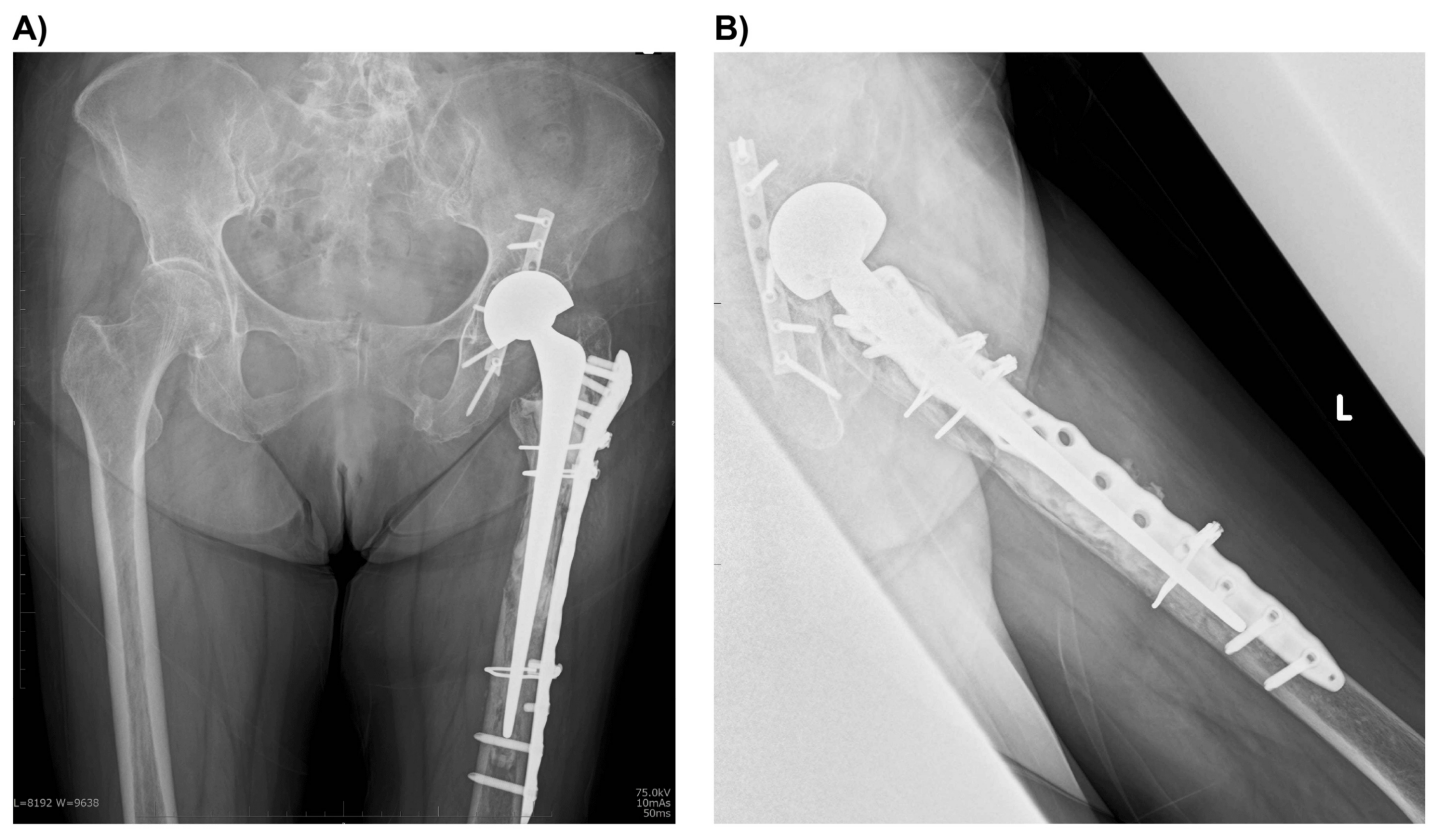
Radiographs at two years postoperatively showing signs of healed fractures. There is no progression of joint gap narrowing, and no evidence of stem subsidence or loosening. (A) Anteroposterior view. (B) Lateral view.

At two years postoperatively, bone fusion had occurred, with no evidence of joint gap narrowing, stem subsidence, or loosening (Figures 6A, 6B). Clinically, the patient’s ADL improved to walking with a single cane, and the Harris Hip Score was 75 points. The patient provided written informed consent for print and electronic publication of this case report.

## Discussion

Recent increases in life expectancy have led to a rise in the number of elderly individuals undergoing procedures such as BHA and THA. Consequently, the incidence of implant-related fractures has also increased and is expected to continue rising. The incidence of periprosthetic fractures associated with THA varies widely, with studies reporting an incidence of 0.1%-18% [2,3].

Evidence suggests that the incidence of PPFFs following bipolar hip arthroplasty is relatively high, approximately 2.4%, even with a relatively short average follow-up period of 44.4 months [4]. This incidence is notably higher after revision arthroplasty. It is anticipated that the frequency of periprosthetic PPFF around hip arthroplasties will continue to rise, making them increasingly common in clinical practice.

A nationwide study in Finland reported a 30% increase in the incidence of acetabular fractures among individuals aged >65 years over the past 20 years, with a current incidence rate of 23 per 100,000 individuals per year [6]. Similarly, Ferguson et al. observed a more than two-fold increase in acetabular fractures among North American patients over the age of 60 during a comparable period. The incidence of acetabular fractures in the elderly is increasing [7]. While high-energy trauma is the primary cause of acetabular fractures in younger patients, most fractures in the elderly result from low-energy trauma, such as falls from standing height [2].

The incidence of occult fractures around the hip joint, including the pelvis, is reported to be 2%-10%. However, the incidence of acetabular fractures specifically is much lower at 0.35%-0.44%, indicating a rare occurrence [5]. Given that elderly individuals often have osteoporosis, they are at increased risk of PPFF following THA. Consequently, there is a significant risk of ipsilateral acetabular insufficiency fractures due to low-energy trauma, such as falls from standing height. When diagnosing fractures, x-rays may not always provide a definitive diagnosis. In such cases, further imaging studies, such as CT scans or MRIs, are conducted to confirm the diagnosis. CT scans are highly effective for evaluating fractures; however, diagnosing occult or non-displaced fractures can still be challenging with CT alone. Therefore, MRI is often used in conjunction to assess surrounding tissue damage and offers a more comprehensive confirmation of the diagnosis. Implants made from ferromagnetic metals can interfere with the magnetic field of MRI, potentially compromising imaging accuracy. This interference may lead to difficulties in detecting fractures around implants, increasing the risk of missed diagnoses on MRI [8]. In CT scans, metal artifacts can also affect the accurate evaluation of the tissue and bone around the implant. Consequently, the possibility of occult fractures should be considered in periprosthetic fractures following BHA and THA.

The Vancouver classification is commonly used to guide the treatment approach for PPFFs. Both classifications share the principle that if the stem is loose, revision surgery is indicated. Conversely, if the implant is well-fixed, open reduction and internal fixation (ORIF) is the preferred treatment [9].

In our case, preoperative x-ray findings revealed a periprosthetic fracture accompanied by cortical bone thinning and cement debonding due to osteolysis, indicating significant prosthesis loosening. Consequently, a revision femoral stem was inserted to bypass the fracture site, while the fracture itself was treated with ORIF.

The treatment options for Vancouver type B3 PPFFs are complex and varied. Typically, a revision femoral stem can be inserted to bypass the fracture site. However, treating B3 fractures is challenging and often requires additional measures such as structural allograft replacement of the proximal femur, an allograft-prosthetic composite revision, a proximal femoral replacement prosthesis, or a custom implant, along with impaction bone grafting [10-12]. Alejandro et al. reported that for periprosthetic fractures around hip HA with stem loosening, revision to HA yielded clinical outcomes comparable to those of revision to THA [13].

ORIF is widely accepted as the treatment of choice for acetabular fractures in younger patients. However, no definitive guidelines exist for managing acetabular fractures in the elderly. Studies have shown that acute THA, with or without prior fixation, provides comparable outcomes to ORIF followed by delayed THA in older patients. Specifically, acute THA offers better Oxford Hip Scores, fewer operations, and equivalent complication rates compared to those of delayed THA [14]. We recommend considering acute THA for patients with risk factors for failure that might necessitate delayed THA, such as dome or roof impaction. When managing acetabular fractures in the elderly, acute THA should be compared with delayed THA following acetabular ORIF. Acute THA for acetabular fractures may require additional implants, such as plates and screws for internal fixation or support rings (e.g., anti-protrusion cage, reinforcement ring) in certain cases [15,16].

In the treatment of acetabular fractures following THA, Callaghan et al. reported that conservative management with protected weight-bearing generally yielded poor outcomes. Specifically, four of six patients who achieved bony union eventually required revision due to cup loosening [17]. Peterson et al. found that 80% of patients treated conservatively for periprosthetic acetabular fractures after THA ultimately required cup revision, suggesting that revision surgery is generally the preferred approach [10]. Masri et al. recommended immediate revision until the bony union is achieved. However, they observed that 77% of patients treated with a cage and 56% of those treated with cementless cups had good outcomes, whereas patients treated with cemented cups had poor outcomes [18].

Evidence suggests that revision using plates, cages, or cementless cups, with or without porous tantalum augments, generally yields more favorable long-term outcomes compared to those of cemented cup revisions for periprosthetic acetabular fractures after THA [19,20]. Similarly, for periprosthetic fractures around BHA, the same management principles should be applied, favoring revision with supplementary fixation devices over purely conservative treatment.

In a small number of cases, preoperative diagnosis may be challenging, and the surgeon might only detect pelvic discontinuity during the operation. Especially after BHA, it is crucial to be vigilant not only for PPFFs but also for the possibility of concurrent fragile acetabular fractures following a fall. In cases of Vancouver B3 fractures with significant periprosthetic osteolysis, the focus may often be on identifying PPFFs, potentially leading to non-displaced acetabular insufficiency fractures being overlooked.

Even in cases of non-displaced acetabular fractures that are difficult to diagnose on CT, displacement can occur during intraoperative manipulation, particularly in patients with severe bone fragility. Therefore, for non-displaced acetabular fractures, it is essential to prioritize acetabular-side bone fixation, including cup placement, before proceeding with further interventions.

Patients who sustain periprosthetic fractures after BHA often have severe bone fragility and a high overall risk. Consequently, it is crucial to perform minimally invasive surgery and facilitate early weight-bearing ambulation postoperatively. In this case, the acetabular fracture was classified as transverse, and posterior wall plating alone may have been inadequate in terms of stability. Therefore, it would have been preferable to also use a cementless cup and support cage for additional fixation. Therefore, the possibility of pelvic discontinuity should be considered when performing a revision THA with PPFF and appropriate prepared measures should be ensured.

However, as in the present case, if ORIF, an anatomical reduction of an acetabular fracture in an elderly patient after BHA is possible, the patient may have a relatively good outcome, albeit in the short term. The choice of cup implantation should be made in consideration of the patient's general condition.

## Conclusions

This case highlights the complexity of managing PPFFs when combined with non-displaced acetabular fractures following BHA. Accurate preoperative diagnosis and intraoperative assessment are essential for effective treatment. Surgeons should consider the possibility of concurrent acetabular fractures in similar cases to optimize surgical outcomes. Future research should focus on improving diagnostic strategies and treatment protocols to better address these rare but challenging fracture patterns.
